# A case report of Sanfilippo syndrome – the long way to diagnosis

**DOI:** 10.1186/s12883-022-02611-7

**Published:** 2022-03-15

**Authors:** Delia Lorenz, Thomas Musacchio, Erdmute Kunstmann, Eva Grauer, Natalie Pluta, Annika Stock, Christian P. Speer, Helge Hebestreit

**Affiliations:** 1 Center for Rare Diseases, University Hospital of Wuerzburg, Josef-Schneider-Strasse 2, 97080 Wuerzburg, Germany; 2 University Children’s Hospital Wuerzburg, University Hospital of Wuerzburg, Josef-Schneider- Strasse 2, 97080 Wuerzburg, Germany; 3 Department of Neurology, University Hospital of Wuerzburg, Josef-Schneider-Strasse 2, 97080 Wuerzburg, Germany; 4 Department of Human Genetics, University of Wuerzburg, Am Hubland, 97074 Wuerzburg, Germany; 5 Department of Neuroradiology, University Hospital Wuerzburg, Josef-Schneider-Strasse 2, 97080 Wuerzburg, Germany

**Keywords:** Mucopolysaccharidosis IIIa, p.R245H, p.S298P, Genotype-phenotype correlation, Diagnostic delay

## Abstract

**Background:**

Mucopolysaccharidosis type III (Sanfilippo syndrome) is a lysosomal storage disorder, caused by a deficiency in the heparan-N-sulfatase enzyme involved in the catabolism of the glycosaminoglycan heparan sulfate. It is characterized by early nonspecific neuropsychiatric symptoms, followed by progressive neurocognitive impairment in combination with only mild somatic features. In this patient group with a broad clinical spectrum a significant genotype-phenotype correlation with some mutations leading to a slower progressive, attenuated course has been demonstrated.

**Case presentation:**

Our patient had complications in the neonatal period and was diagnosed with Mucopolysaccharidosis IIIa only at the age of 28 years. He was compound heterozygous for the variants p.R245H and p.S298P, the latter having been shown to lead to a significantly milder phenotype.

**Conclusions:**

The diagnostic delay is even more prolonged in this patient population with comorbidities and a slowly progressive course of the disease.

## Background

Mucopolysaccharidosis type III (MPS III), also known as Sanfilippo syndrome [1], is considered as the most common type of MPS [2]. The four subtypes (A-D) are characterized by different enzymatic deficiencies due to the underlying affected gene and therefore by accumulation of undegraded glycosaminoglycans leading to tissue damage. Within MPS III group type IIIa is the most common and severe type with earliest onset, rapid clinical course and short survival [2]. MPS IIIa has an autosomal-recessive trait and is caused by mutations in the SGSH gene on chromosome 17q25.3 leading to heparan-N-sulfatase deficiency. The clinical phenotype of MPS III is characterized by a severe degeneration of the central nervous system, starting with a symptom-free interval, first symptoms between 2 and 6 years, going on with slow loss of skills at the end of first decade of life and ending with a vegetative state or death generally in the second or third decade. The rate of progression is highly variable and seems to be partly related to the genotype. Diagnostic delay is common because of gradual onset, highly variable clinical course, lack of specific somatic features and limitations of sensitive laboratory indices. It is even more prolonged in patients with comorbidities and / or a slowly progressing phenotype.

## Case presentation

The parents of a 28 years old patient contacted our centre for rare diseases to search for a diagnosis in respect of a severe progressive developmental regression of their son.

### Clinical description

The patient is the first child of non-consanguineous German parents. He has two younger healthy siblings. Pregnancy was normal. Delivery was in the 41st week by an emergency caesarean section with APGAR 3/8/9 and green amniotic fluid. Birth weight was 3600 gram, height 51 cm and head circumference 36.5 cm. The neonate had to be ventilated for one day. At day 7 he suffered from an acute renal failure and needed peritoneal dialysis. The underlying cause of this condition is unknown, renal function was unimpaired thereafter. He was discharged from hospital at the age of 6 weeks.

Motor development was normal in his first year (walking with 13 months). Up to adolescence, he could walk unassisted and learned cycling at the age of 12 years. Fine motor skills were always slightly impaired. Mental and speech development was delayed. He was diagnosed with global developmental delay at the age of 2 years. At the age of 4–5 years, he could speak 5-word sentences. Regression of motor skills, speech and cognitive abilities started after the age of 12 years. With 16 years he was diagnosed with a scoliosis, needed an orthopaedic corset, and later on at the age of 27 years a spondylodesis. He lost the ability to walk free in early adulthood. At the age of 16 years, he could only speak 1 to 2 word combinations, complete loss of speech was at the age of 18 years. The patient had recurrent ear infections in infancy and childhood. An audiogram was normal at the age of 2 years, at the age of 9 years he needed a hearing aid due to sensorineural hearing loss. Somewhere along the course of the disease, visual impairment occurred - the starting point of this impairment is not clear. High-grade hearing and visual impairment have been reported since the age of 24 years. The patient is now fully dependent on assistance for all activities of daily living.

Previous diagnostic workup included developmental evaluation at the ages of 3 and 7 years. Metabolic diagnostics at the age of 3 years showed normal results for amino acids, organic acids and long chain saturated fatty acids, carnitin, acylcarnitin, purine and pyrimidine. There is no information on a urinary Glycosaminoglycan level. Psychiatric evaluation at the age of 14 years revealed behavioural abnormalities, restlessness, hyperkinetic disorder, stereotypies and mental retardation.

The current clinical examination showed a conscious patient, who was not able to make contact with other people. The patient had no distinct facial features and no hepatosplenomegaly at physical examination. He showed continuous vocalizations, severely impaired vision and hearing, a spasticity of the extremities and enhanced reflexes. He could stand and walk a few steps with assistance. Ophthalmologic examination revealed a partial optic nerve atrophy, Brainstem Electric Response Audiometry showed a high-grade hearing impairment / hearing loss.

### Genetic analysis

Genomic DNA was extracted from peripheral blood leukocytes of the patient and whole exome sequencing was performed. Library preparation was performed with the Nextera Rapid Capture Exome Kit (Illumina). Sequencing was done on a NextSeq desktop sequencer (Illumina). Data were analyzed with GensearchNGS (PhenoSystems SA) and interpreted with Alamut Visual (Interactive Biosoftware). Filtering and prioritizing of variants were performed by using a phenotype-based approach and quality criteria.

### Radiologic features

**Fig. 1:**
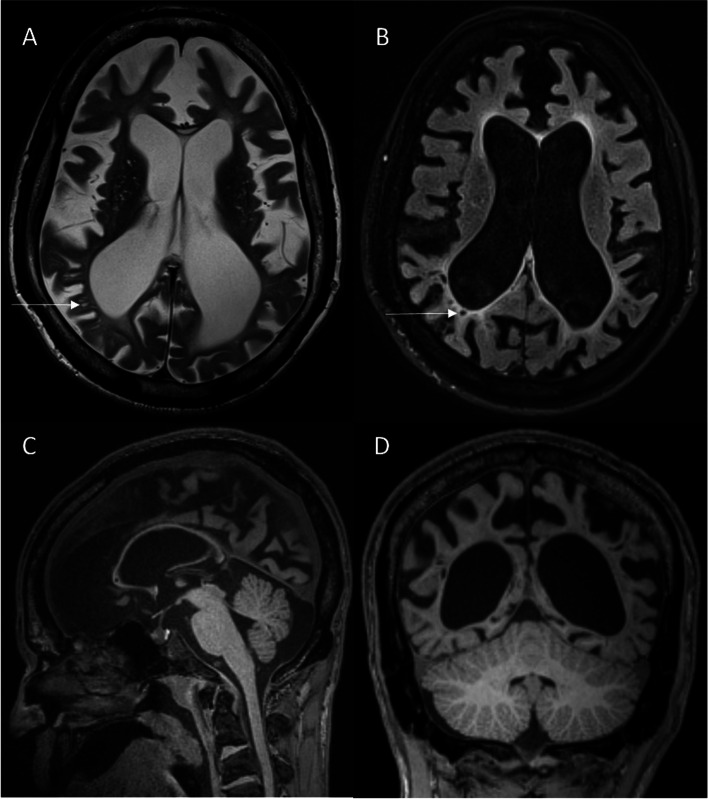
Brain MRI at the age of 28 years. Transversal T2-weighted image (**A**) shows ventriculomegaly and enlarged cortical sulci due to brain atrophy. There is associated enlargement of the perivascular spaces (arrows). **B** Fluid-attenuated inversion recovery (FLAIR) sequence shows only a slight periventricular increase in signal intensity, which can be attributed to hypothetical dysmyelination. On 3D-T1-weighted images, sagittal reconstruction (**C**) demonstrates a thin corpus callosum and coronal reconstruction (**D**) shows the convincingly reduced volume of the supratentorial hemispheres, whereas the cerebellum appears normal

The first brain MRI at the age of 28 years demonstrated a significant loss in volume of the hemispheres, predominantly of the white matter. Due to the loss in volume, ventriculomegaly, enlarged perivascular spaces and cortical sulci were present. The corpus callosum was thinned out. Hypothetically, dysmyelination led to the narrow-banded increase in T2-signalintensity of the periventricular white matter, while the majority of the white matter was spared. The imaging changes were limited to the supratentorial level. The cerebellum appeared of normal size (Fig. 1).

## Results

Two heterozygous variants in the gene SGSH (hg19:NM_000199.3) were identified: c.734G > A, p.R245H and c.892T > C, p.S298P. Both variants can be classified as pathogenic by ACMG/AMP 2015 guidelines [3] and are reported as pathogenic in various databases (e.g. OMIM, ClinVar, HGMD). Sanger sequencing of the parents verified the compound heterozygosity of the identified variants and thus confirmed autosomal recessive inheritance of mucopolysaccharidosis type IIIa.

## Discussion and conclusion

A total of 142 disease-causing mutations (Human Gene Mutation Database, 2019/4) have been described to date, most of them (77%) are missense mutations. The variant c.734G > A, p.R245H is the most common pathologic mutation within the SGSH-gene in northern Europe. It leads to a nearly complete loss of sulfamidase activity. In a homozygous state, it is associated with rapid and severe disease progression. The c.892T > C, p.S298P mutation shows a milder course of the disease. Compound heterozygous patients with both variants, as in our patient, have been reported with a mild and attenuated course [4].

A large Dutch study on 111 patients with MPS IIIa reported allelic frequencies of p.R245H and p.S298P of 44.9% and 18.6% respectively. Twenty-three patients (23%) had the combination of both mutations [5]. Within the highly heterogeneous phenotype of MPS IIIa the rate of disease progression is related to variations in residual enzyme activity and allelic heterogeneity and shows strong genotype-phenotype correlations. Some mutations or combination of mutations are associated with a severe phenotype (p.R245H, p.Q380R, p.566W), whereas other variants (p.S298P, p.G122R, p.R206P, p.I322S, p.E369K) are associated with an intermediate or mild, attenuated course of the disease [4–8]. Patients, who are compound heterozygous for p.S298P in combination with one of the severe mutations (e.g. p.R245H), also showed a milder phenotype [5]. The milder course within these patients may be due to a residual sulfamidase activity of up to 3 to 11% of normal controls [9, 10].

The so far described clinical phenotype of patients with the same compound heterozygous mutations of p.R245H and p.S298P is similar to the clinical course of our patient. In this patient group, the median age at loss of speech was 15y and the median age at loss of independent walking was 25y. The reported median age of death was 33.5 to 38y [4, 5]. Diagnosis of patients with a slowly progressive course of the disease was on average 5y later than in patients with rapid progression [6, 11].

Radiologic features in mucopolysaccharidosis comprise focal or diffuse, characteristically periventricular or bilateral T2-hyperintensity, enlarged periventricular spaces, involvement of the corpus callosum and brain atrophy. Hydrocephalus is more frequent in other types of mucopolysaccharidosis. In our patient, the most obvious abnormalities were a significant loss in volume and enlarged periventricular spaces, whereas increased T2-signalintensity was limited to a narrow band of the periventricular white matter. The severity of mental retardation has been shown to be associated with the severity of neuroimaging anomalies [2, 12].

The history of complications during delivery and in early neonatal life may have contributed to the delayed recognition of MPS IIIa in our patient. Developmental delay was initially seen as a consequence of perinatal asphyxia. Early, non-specific symptoms as behavioural abnormalities, hyperactivity and sleep disturbances may mislead to diagnoses of attention deficit / hyperactivity disorder and / or autism spectrum disorders [11]. Somatic features are usually mild and non-specific and therefore not indicative in the diagnostic process. Patients with MPS IIIa are often diagnosed by paediatricians specialized in the broad field of metabolic disorders. Patients with a slowly progressing phenotype may have less contact with specialized paediatricians and consult doctors later in adolescence or early adulthood when neurodevelopmental regression is becoming obvious. Diagnostic delay may thus be due to a limited awareness of metabolic disorder spectrum in adult patients.

MPS III is a diagnostic challenge, particularly in the early stages and in patients with an attenuated course of the disease, due to a variable course, nonspecific early neuropsychiatric symptoms and the lack of obvious somatic features. The possibility of a metabolic disorder should be kept in mind in young children with developmental and / or speech delay in combination with behavioural abnormalities and / or sleeping difficulties and in adults, especially when pre-existing health conditions may obscure the clinical picture.

## Data Availability

The datasets used and/or analyzed during the current study are available from the corresponding author on reasonable request.

## Abbreviations

MPS: Mucopolysaccharidosis
DNA: Deoxyribonucleic acid
MRI: Magnetic resonance imaging
